# Repair for Mitral Valve Aneurysm using autologous pericardium: a case of our experience

**DOI:** 10.1186/s13019-014-0148-y

**Published:** 2014-09-18

**Authors:** Hongqiang Zhang, Hao Chen, Xiaoning Sun, Shouguo Yang, Chunsheng Wang

**Affiliations:** Department of Cardiac Surgery, Zhongshan Hospital of Fudan University, No. 180 Fenglin Road, Xuhui District, Shanghai, 200032 China

**Keywords:** Mitral valve aneurysm, Mitral valve plasty

## Abstract

Mitral Valve Aneurysm (MVA) is rarely reported and occurs most in association with infective endocarditis of the aortic valve. In our case, the 46-yr-old Chinese man was referred to our hospital with dyspnea and orthopnea. Transesophageal echocardiography during operation revealed a localized, thin-walled saccular structure in the anterior leaflet that bulged into the left atrium, and severe mitral and aortic regurgitation. Aortic valve was replaced with mechanical prostheses and mitral valve was repaired with autologous pericardium. Transesophageal echocardiography during operation and transthoracic echocardiography 3 months later showed mild regurgitation.

**Electronic supplementary material:**

The online version of this article (doi:10.1186/s13019-014-0148-y) contains supplementary material, which is available to authorized users.

## Background

Mitral Valve Aneurysms (MVA) are rarely reported, and often occur as complications of infective endocarditis of the aortic valve. The case was referred to our hospital, with MVA and severe aortic regurgitation, without any evidence for active endocarditis. After perioperative preparation, Aortic valve was replaced with mechanical prostheses and mitral valve was repaired with autologous pericardium. Transesophageal echocardiography during operation and transthoracic echocardiography 3 months later show mild regurgitation.

## Case presentation

**Figure 1:**
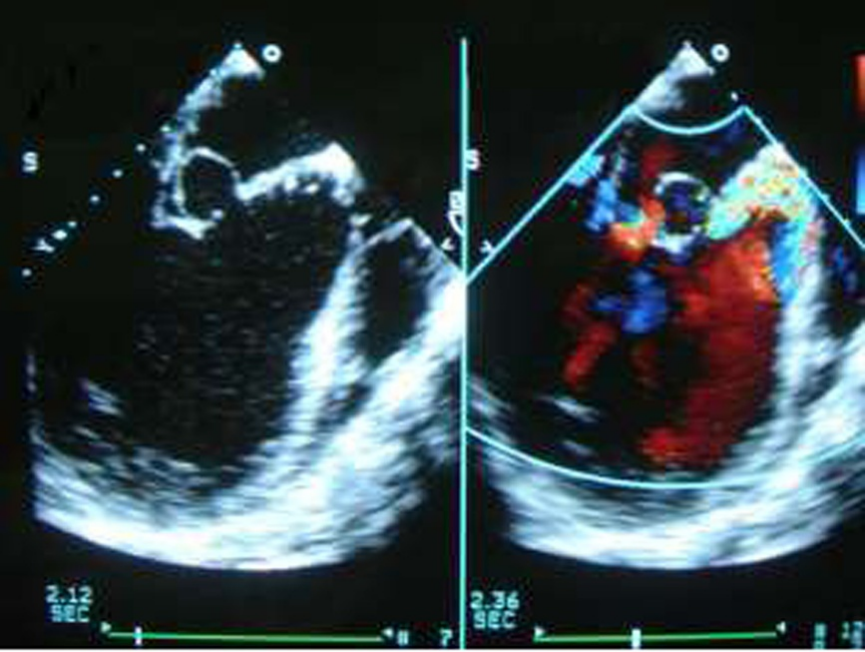
**Transesophageal echocardiography during operation revealed a saccular structure in the anterior leaflet that bulged into the left atrium throughout the cardiac cycle.**

A 46-yr-old Chinese man was referred to our hospital, with dyspnea and orthopnea. Two months before this, he was admitted to another hospital for suffering from high fever (39.2°C). Large doses of penicillin were intravenously given then because of positive blood culture, which showed Viridans Streptococci. He came to our hospital because of shortness of breath and orthopnea, but no fever for 40 days. Transesophageal echocardiography during operation revealed a saccular structure in the anterior leaflet that bulged into the left atrium throughout the cardiac cycle (Figure 1), and also the anterior leaflet of the mitral valve was redundant and prolapsed due to the aneurysm. Severe aortic regurgitation was detected on TEE, which led to the enlargement of left ventricle and mitral annulus. Physical examination found diastolic murmur on the left sternal border and systolic murmur at the apex. Infective endocarditis was suspected, so after admission blood cultures were repeated which were all negative, also medical therapy for heart failure was started and resulted in clinical stabilization rapidly.

**Figure 2:**
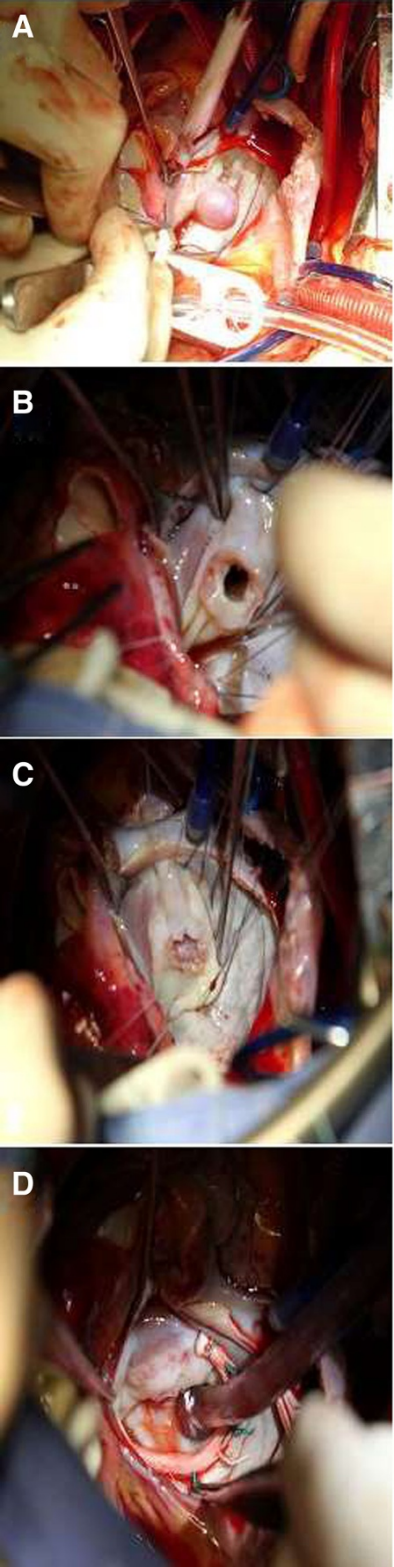
**How to deal with Mitral Valve Aneurysm during operation. A**: Showing MVA from the left atrium side. **B**: After resection of the MVA. **C**: Repairing using autologous pericardium. **D**: Putting the prosthetic ring.

The patient underwent aortic valve replacement with 23 mm bileaflet mechanical prostheses (St. Jude Medical) and mitral valve plasty with autologous pericardium. At operation, no vegetation was found on the coronary cusp, the aortic valve appeared to be thicken and coarse, but no vegetation. A cystic cavity of 15 mm on the anterior mitral leaflet was clearly visualized from the left atrium and was unbroken (Figure 2(A)). The mitral valve leaflet was smooth and no visible evidence of endocarditis was found. Therefore we just resect the MVA at its bottom and the aortic valve as normal. After resection of the MVA, an oval deficit of 6 mm in diameter on the anterior mitral leaflet was visualized (Figure 2(B)), which was repaired with autologous pericardium (10 × 10 mm) as usual, and the patch was placed at the left ventricle side of the valve through left atrium (Figure 2(C)). The autologous pericardium patch was excised, treated with 0.625% glutaraldehyde solution for 10 minutes, and rinsed with physiologic saline. Also a 30 mm C Type Sorin prosthetic ring was used for mitral annuloplasty due to the enlargement of the mitral annulus (Figure 2(D)). Transesophageal echocardiography during operation showed mild regurgitation. After operation, although cultures of the removed aortic leaf-lets and MVA were negative and further pathology showed no evidence of inflammatory infiltrate, which was due to the administration of enough doses of antibiotics before the operation, vancomycin was still intravenously given to prevent the recurrence of infection (1.0 g q12 h for 2 weeks). The patient recovered uneventfully and was discharged asymptomatic on the tenth postoperative day. 3 months later transthoracic echocardiography still showed a perfect result.

## Discussion

Mitral Valve Aneurysm (MVA) was first described in 1729 by Morand and frequently found in the anterior leaflet. However the mechanism of its formation is not clear now because of rare occurrence, the etiology is believed to be related to aortic valve endocarditis but it’s not a complication of myocardial necrosis [1]-[3]. Two reasons may be responsible. First, the infection from the aortic valve may spreads to the mitral aortic intervalvular fibrosa and also the infected aortic regurgitant jet striking the ventricular surface of the anterior mitral leaflet can result in the formation of an aneurysm. Second, there may be a congenital defect of the fibrous layer of the atrioventricular junction, which plays an important role in the formation of an aneurysm. In our case, the first reason seems to be proved. There are literatures reporting MVA forming in patients without endocarditis, which support the second reason. While these cases usually have connective tissue disorders, myxomatous valvular degeneration, Marfan syndrome, pseudoxanthoma elasticum, or physical stress due to severe aortic regurgitation [2].

Uncommon MVA were mostly diagnosed by echocardiographic findings, and often described as a localized, thin-walled saccular bulge of the mitral leaflet toward the left atrium associated with systolic expansion and diastolic collapse [4],[5]. Its clinical presentation varies from no symptoms to sudden death, which is related to any of the preceding manifestations and occasionally due to spontaneous rupture of the aneurysm. So Early detection and prompt intervention are important to prevent the complications of valvular aneurysms which include rupture and embolism.

Surgical treatment including replacement or repair is indicated when aneurysm ruptures or when the unruptured aneurysm is larger or accompanied by significant regurgitation [5]. For cases suffering from endocarditis, antibiotics therapy is necessary before surgery until the patient’s temperature fluctuated for at least 4 weeks and blood culture is negative. In our case, the patient has no fever for 40 days before surgical treatment, cultures of the removed aortic leaf-lets and MVA were negative and further pathology showed no evidence of inflammatory infiltrate, which is important for future. The mitral valve can be repaired with autologous pericardium [5] as in our case, make sure that the autologous pericardium is placed at the left ventricle side and also close follow-up is needed.

## Conclusions

In patients with Mitral Valve Aneurysm, transthoracic echocardiography or transesophageal echocardiography is mandatory to make a diagnosis and Reparing for Mitral Valve Aneurysm using autologous pericardium is a good choice when indicated.

## Consent

Written informed consent was obtained from the patient for publication of this case report and any accompanying images. A copy of the written consent is available for review by the Editor-in-Chief of this journal. This study was approved by the Institutional Review Board of Zhongshan Hospital, Fudan University.

## Authors’ original submitted files for images

Below are the links to the authors’ original submitted files for images. Authors’ original file for figure 1 Authors’ original file for figure 2

## Abbreviation

MVA: Mitral Valve Aneurysm
