# Supplementary figures and images for: Repair for Mitral Valve Aneurysm using autologous pericardium: a case of our experience
Source: J Cardiothorac Surg. 2014 Sep 18;9:148. doi: 10.1186/s13019-014-0148-y (PMC4182794; doi:10.1186/s13019-014-0148-y)

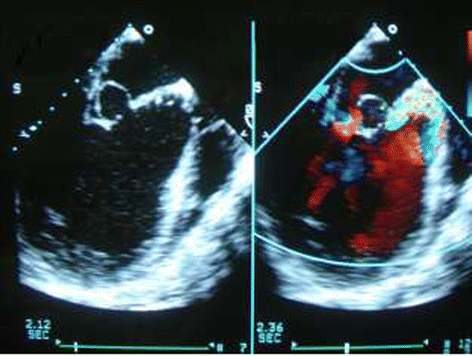

Supplement: Supplementary file 1 — Authors’ original file for figure 1 [file 13019_2014_148_MOESM1_ESM.gif]

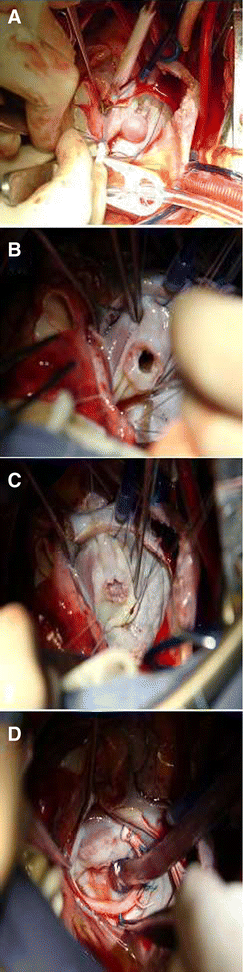

Supplement: Supplementary file 2 — Authors’ original file for figure 2 [file 13019_2014_148_MOESM2_ESM.gif]
